# The pericyte as a cellular regulator of penile erection and a novel therapeutic target for erectile dysfunction

**DOI:** 10.1038/srep10891

**Published:** 2015-06-05

**Authors:** Guo Nan Yin, Nando Dulal Das, Min Ji Choi, Kang-Moon Song, Mi-Hye Kwon, Jiyeon Ock, Anita Limanjaya, Kalyan Ghatak, Woo Jean Kim, Jae Seog Hyun, Gou Young Koh, Ji-Kan Ryu, Jun-Kyu Suh

**Affiliations:** 1 National Research Center for Sexual Medicine and Department of Urology, Inha University School of Medicine, Incheon 400-711, Republic of Korea; 2 Department of Urology, Gyeongsang National University School of Medicine, Jinju 660-702, Republic of Korea; 3 Department of Biological Sciences and Laboratory for Vascular Biology, Korea Advanced Institute of Science and Technology (KAIST), Daejeon 305-701, Republic of Korea; 4 Inha Research Institute for Medical Sciences, Inha University School of Medicine, Incheon 400-711, Republic of Korea

## Abstract

Pericytes are known to play critical roles in vascular development and homeostasis. However, the distribution of cavernous pericytes and their roles in penile erection is unclear. Herein we report that the pericytes are abundantly distributed in microvessels of the subtunical area and dorsal nerve bundle of mice, followed by dorsal vein and cavernous sinusoids. We further confirmed the presence of pericytes in human corpus cavernosum tissue and successfully isolated pericytes from mouse penis. Cavernous pericyte contents from diabetic mice and tube formation of cultured pericytes in high glucose condition were greatly reduced compared with those in normal conditions. Suppression of pericyte function with anti-PDGFR-β blocking antibody deteriorated erectile function and tube formation *in vivo* and *in vitro* diabetic condition. In contrast, enhanced pericyte function with HGF protein restored cavernous pericyte content in diabetic mice, and significantly decreased cavernous permeability in diabetic mice and in pericytes-endothelial cell co-culture system, which induced significant recovery of erectile function. Overall, these findings showed the presence and distribution of pericytes in the penis of normal or pathologic condition and documented their role in the regulation of cavernous permeability and penile erection, which ultimately explore novel therapeutics of erectile dysfunction targeting pericyte function.

The penis has a specialized vascular bed and erectile dysfunction (ED) is predominantly a disease of vascular origin^1^. Physiologic penile erection requires interaction between vascular endothelial cells (ECs) and smooth muscle cells (SMCs) in the corpus cavernosum. Functional and structural derangements of these cells play a critical role in the pathophysiology of ED from various causes, such as diabetes and cavernous nerve injury^2^,^3^,^4^. These observations paved the way to the development of new treatment modalities targeting regeneration of cavernous ECs and SMCs.

Pericytes were discovered as a population of contractile cells surrounding the ECs of microvessels (arterioles, capillaries, and venules) and were historically defined by their association with capillary ECs. Their presence has been confirmed in a variety of organs and tissues^5^. Pericytes are known to play critical roles in vascular development and cardiovascular homeostasis, such as in endothelial proliferation or differentiation^6^,^7^; in the regulation of vascular contractility, tone, and permeability^8^; and as a potential reservoir of mesenchymal stem cells or progenitor cells^9^. In addition to ECs and SMCs, pericytes are also important for vascular maturation by direct contact or communication with ECs, thereby recruitment of SMCs^10^. By contrast, pericyte loss or dropout is a major pathologic feature of diabetic retinopathy, which leads to capillary leakage and macular edema^11^. Moreover, a recent study in an animal model of myocardial infarction showed that intramyocardial transplantation of human pericytes enhances angiogenesis and improves heart function^12^. Whereas, the distribution of pericytes in the penis and their functional roles in penile erection are as yet largely unknown, with the exception of two reports showing that the sinusoidal endothelium is not associated with pericytes^13^,^14^. However, those studies were ultrastructural evaluations by electron microscopic study and lacked of immunohistochemical studies with specific markers for pericytes. Therefore, localization of pericytes and determination of their roles in the penis will enhance our understanding of pericyte-mediated pathophysiology of erectile function/dysfunction and therapeutic target for ED.

In the present study, we for the first time determined the differential distribution of pericytes and their anatomical relationships with ECs and SMCs in the mouse and human penis by using immunohistochemical staining with three-dimensional reconstruction. We further confirmed the presence of pericytes in the mouse penis with primary cell culture. Moreover, we compared the expression of pericytes in the penis between normal and diabetic mice. Finally, we examined the functional role of pericytes in penile erection by enhancing pericyte expression with intracavernous injection of recombinant human-hepatocyte growth factor (rh-HGF) protein into diabetic mice and by suppressing pericyte function with intracavernous injection of anti-platelet-derived growth factor receptor-β (PDGFR-β) blocking antibody into normal mice.

## Results

### Localization of pericytes in the penis of normal mice

**Figure 1:**
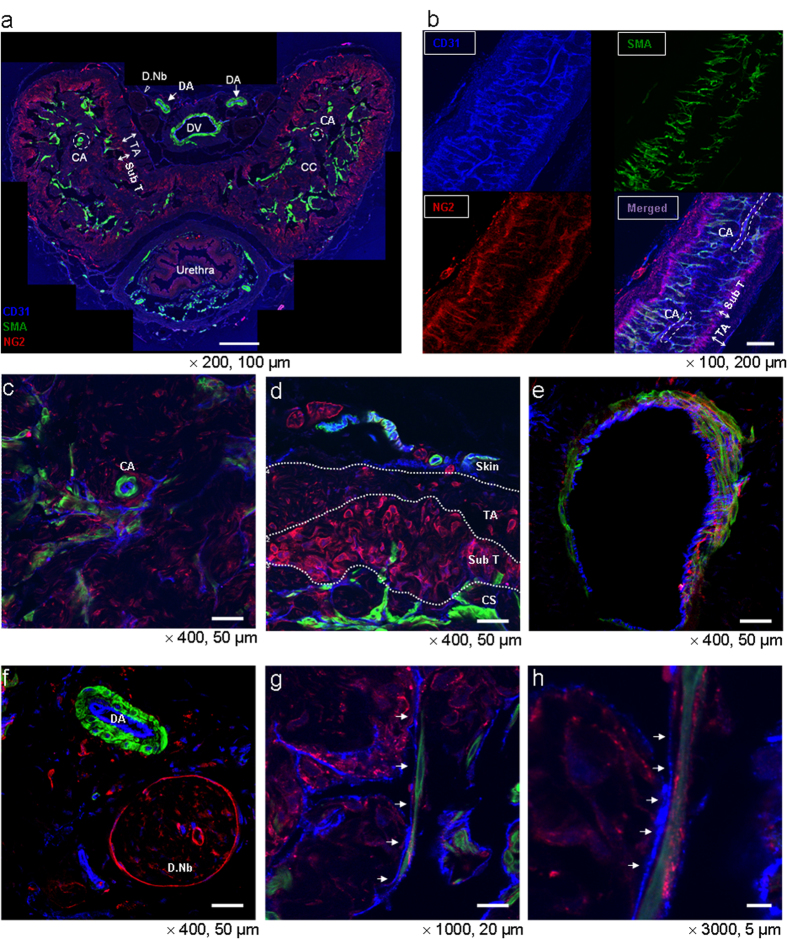
Distribution of pericytes in the penis of normal mice: low and high magnification images. (**a**) Merged image of transverse thin-cut (7 μm) sections. Immunofluorescent triple staining of penile tissue performed with antibodies against CD31 (an endothelial cell marker, blue), smooth muscle α-actin (SMA, a smooth muscle cell marker, green), and NG2 (a pericyte marker, red) in a 12-week-old male mouse. Scale bar = 200 μm and screen magnification = × 200. (**b**) Representative z-stacks of longitudinal thick-cut (50 μm) sections depicted by CD31, smooth muscle α-actin, and NG2 staining. Scale bar = 200 μm and screen magnification = ×100. **(c**-**h)** Merged images of transverse thick-cut (50 μm) sections depicted by CD31, smooth muscle α-actin, and NG2 staining. **(c)** Cavernous sinusoids and cavernous artery. **(d)** Defined image of subtunical area and tunica albugenia with white dotted demarcation. **(e)** Dorsal vein. **(f)** Dorsal artery and dorsal nerve bundle. Scale bar = 50 μm and screen magnification = ×400 for a, b, c, d. **(g, h)** Higher magnification images of cavernous sinusoids. Scale bar = 20 and 5 μm and screen magnification = ×1000 and ×3000, respectively for **g** and **h**. Images are representative of four-independent experiments. Different penile areas are marked by arrows or specific demarcation. CA = cavernous artery; CC = corpus cavernosum; CS = cavernous sinusoids; DA = dorsal artery; D.Nb = dorsal nerve bundle; DV = dorsal vein; TA = tunica albuginea; Sub T = subtunical area.

Immunofluorescent triple staining of penile cavernous tissue was performed with antibodies against CD31 (an EC marker), smooth muscle α-actin (SMA, an SMC marker), and NG2 (a pericyte marker). We analyzed both the thin-cut (7-μm) and thick-cut (50-μm) transverse or longitudinal sections through low to high-magnification confocal images. The low-magnification images revealed that ECs and SMCs were evenly and abundantly distributed throughout the erectile tissue, whereas pericytes were mainly located in the periphery of the erectile tissue, especially in the subtunical area of corpus cavernosum (Fig. 1a,b). The high-magnification images also confirmed the most intense and abundant expression of NG2-positive pericytes around microvessels at the subtunical area, followed by the dorsal nerve bundle, dorsal vein, and cavernous sinusoids. The endothelial layer of the cavernous artery or dorsal artery was mainly covered with SMCs and rarely overlapped with pericytes (Fig. 1c–h).

To further delineate the anatomical relationships between ECs, SMCs, and pericytes, we reconstructed 3-D images of the transverse sections subjected to immunofluorescent staining (see Supplemental Fig. 1 online). In individual sections and stacked image of the cavernous sinusoids, CD31-positive ECs, SMA-positive SMCs, and NG2-positive pericytes composed sinusoidal or vascular wall. Pericytes and SMCs were found to cover the endothelial layer, facing the lumen of the wall (see Supplemental Fig. 2a, b online). Histologically, pericytes were abundantly distributed in microvessels of the subtunical area and dorsal nerve bundle of mice, followed by dorsal vein and cavernous sinusoids (see Supplemental Fig. 2c–h online). In contrast, relatively large arteries, including cavernous and dorsal artery, showed almost absence of pericytes (see Supplemental Fig. 2i, j online). We also confirmed such observations through the live 3-D video images for each area, which are available in the *Online Supplementary Information* (see Supplemental Video Files 1-5 online).

### Localization of pericytes from the human corpus cavernosum tissue

**Figure 2:**
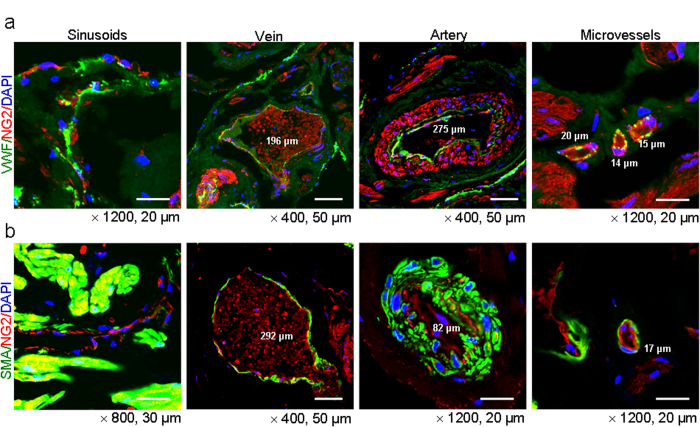
Localization of pericytes in the penis of congenital penile curvature patients with normal erectile function. Merged image of transverse thin-cut (7 μm) sections. **(a)** Immunofluorescent double staining of penile tissue performed with antibodies against VWF (an endothelial cell marker, green) and NG2 (a pericyte marker, red). DAPI = 4,6-diamidino-2-phenylindole (a nuclei marker, blue). Scale bar = 20 μm or 50 μm. **(b)** Immunofluorescent souble staining of penile tissue performed with antibodies against smooth muscle α-actin (SMA, a smooth muscle cell marker, green) and NG2 (a pericyte marker, red). DAPI (a nuclei marker, blue). Scale bar = 20 μm, 30 μm, or 50 μm. Images are representative of four independent samples.

Immunofluorescent double staining of human cavernous tissue with antibodies against VWF (an EC marker) and NG2 revealed that the EC layer was covered with NG2-positive pericytes in sinusoids, as well as vein, artery, and microvessels (Fig. 2a). The smooth muscle α-actin and NG2 double staining revealed that some portion of NG2-positive cells is overlapped with α-actin-positive cells (Fig. 2b). This finding is similar to results from previous study showing that NG2 proteoglycan is expressed exclusively by mural cells^15^.

### Isolation of pericytes from mouse corpus cavernosum tissue

**Figure 3:**
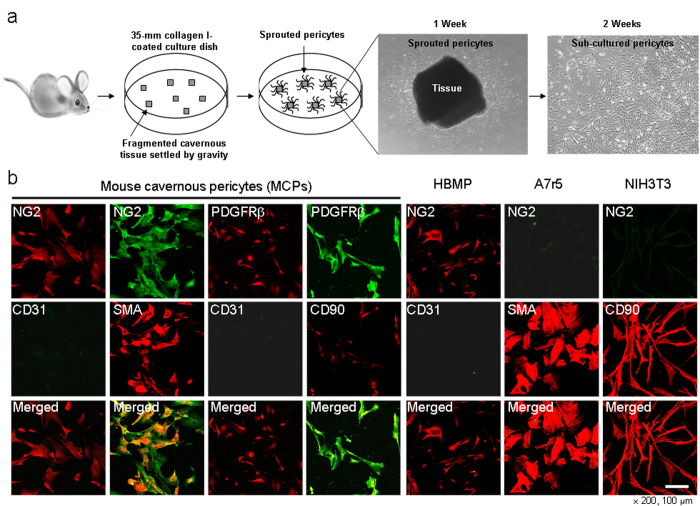
Schematic representation of the primary culture for mouse cavernous pericytes (MCPs). (**a**) The corpus cavernosum tissue was implanted on a collagen I-coated 35-mm cell culture dishes with pericyte culture medium. After cells were confluent and spread on the whole bottom, only sprouting cells were used for subcultivation. (**b**) Characterization of MCPs by fluorescent immunocytochemistry with antibodies against NG2, PDGFR-β (makers for pericytes), CD31, smooth muscle α-actin (SMA), and CD90. Scale bar = 100 μm and screen magnification = ×200. Human microvascular pericytes (HBMP) were used as a positive control, and A7r5 and NIH3T3 cells were used as negative controls. The results were similar from more than ten-independent experiments. A7R5 = Rat aorta smooth muscle cells; NIH3T3 = Mouse embryonic fibroblasts.

A schematic diagram of the procedure used to isolate the mouse cavernous pericytes (MCPs) is illustrated in Fig 3a. Primary cultured cells showed very similar morphology and highly positive staining for the pericyte markers (NG2 and PDGFR-β)^16^, and also revealed positive staining for α-actin and CD90 (a fibroblast marker), but did not show positive staining for the EC marker CD31. We used human brain microvascular pericytes (HBMP) as a positive control, and A7r5 SMCs and NIH3T3 fibroblasts as negative controls (Fig. 3b).

### Pericyte expression and function are attenuated *in vivo* and *in vitro* diabetic condition

**Figure 4:**
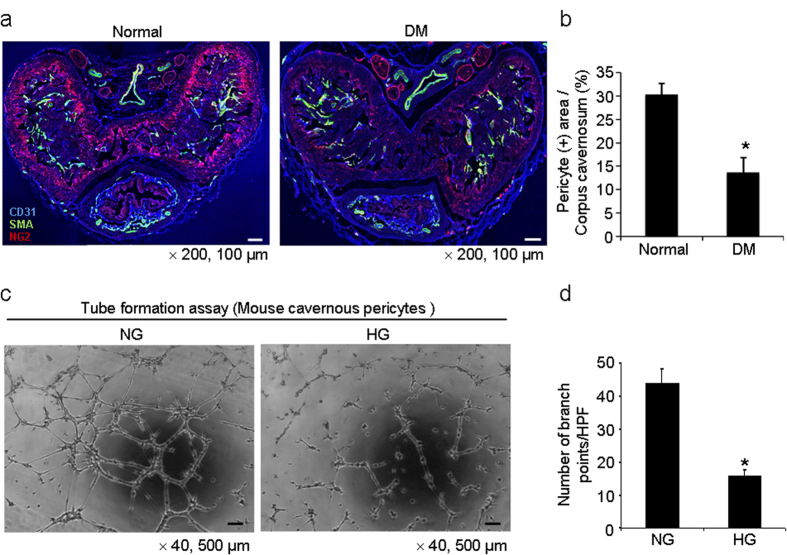
Decrease in pericyte content in the penis of diabetic mice. (**a**) Merged images of transverse thin-cut (7 μm) sections. Immunofluorescent triple staining of penile tissue performed with antibodies against CD31 (blue), smooth muscle α-actin (SMA, green), and NG2 (red) in normal and diabetic mice. Scale bar = 100 μm and screen magnification = ×200. **(b)** An image analyzer was used to quantitate the NG2-immunopositive pericyte area in each group. Each bar depicts the mean ± standard deviations from *n* = 6 animals per group. *P < 0.01 vs. the normal group. DM = diabetes mellitus. (**c**) Tube formation assay in MCPs exposed to normal glucose (NG, 5 mmol) or high glucose (HG, 30 mmol) condition for 48 hours. Scale bars = 500 μm and screen magnification = ×40. **(d)** Number of branch points per high-power field from *n* = 4 wells per group. *P < 0.01 vs. the NG group.

Immunohistochemical triple staining of penile tissue with antibodies against CD31,smooth muscle α-actin, and NG2 was performed in age-matched controls and STZ induced diabetic mice. We observed a profound decrease in pericyte content in the penis of diabetic mice compared with that in the control mice (Fig. 4a,b). Tube formation assay showed decreased branch points of MCPs exposed to high glucose-condition (30 mmol) for 48 hours (Fig. 4c,d).

### Suppression of pericyte function with anti-PGDFR-β blocking antibody decreases erectile function in normal mice *in vivo* and tube formation *in vitro*

**Figure 5:**
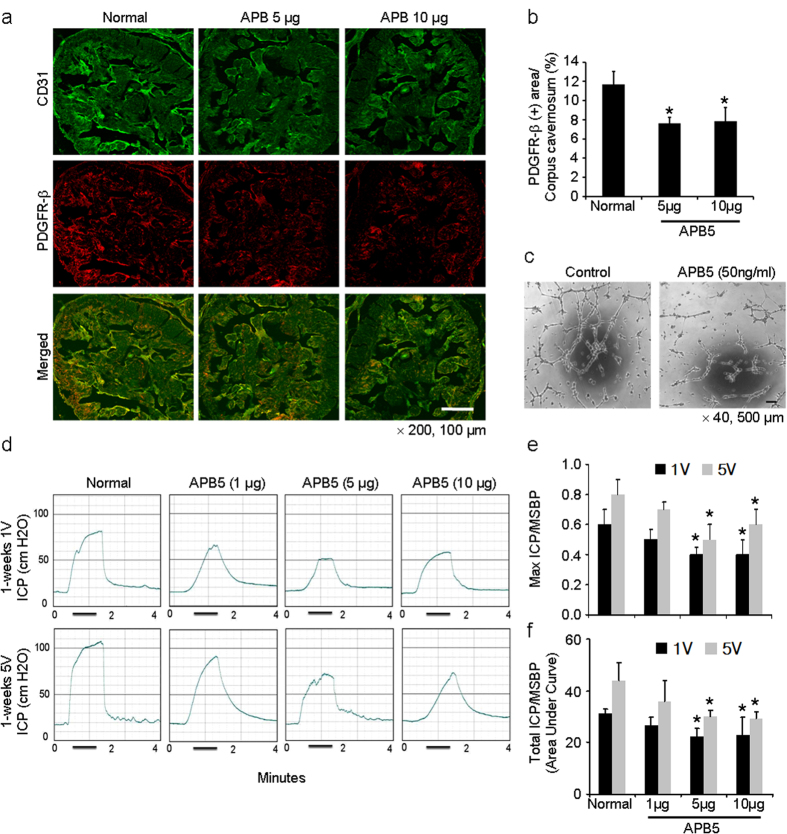
APB5, an anti-PGDFR-β blocking antibody, deteriorates erectile function in normal mice in vivo and pericyte function in vitro. (**a**) Immunofluorescent double staining of cavernous tissue with antibodies to CD31 (green) and PDGFR-β (red) in untreated normal mice or normal mice 1 week after receiving a single intracavernous injection of PBS (20 μl) or APB5 (5 μg/20 μl or 10 μg/20 μl, respectively). Scale bar = 100 μm and screen magnification = ×200. (**b**) An image analyzer was used to quantitate the PDGFR-β-immunopositive area. Each bar depicts the mean ± standard deviation from *n *= 6 animals per group. *P < 0.01 vs. untreated normal group. (**c**) Tube formation assay in MCPs exposed to IgG control or APB5 (50 ng/ml) for 24 hours. Scale bars = 500 μm and screen magnification = ×40. (**d**) Representative intracavernous pressure (ICP) responses for untreated normal mice or normal mice stimulated at 1 week after receiving a single intracavernous injection of PBS (20 μl) or APB5 (1 μg/20 μl, 5 μg/20 μl, or 10 μg/20 μl, respectively). The cavernous nerve was stimulated at 1 and 5 V. The stimulus interval is indicated by a solid bar. (**e**, **f**) Ratios of mean maximal ICP and total ICP (area under the curve) to mean systolic blood pressure (MSBP) were calculated for each group. Each bar depicts the mean ± standard deviation from *n* = 6 animals per group. *P < 0.05 vs. untreated normal group. PDGFR-β, platelet-derived growth factor receptor-beta.

We determined the effect of suppression of pericyte function on erectile function in normal mice by using anti-PGDFR-β blocking antibody, APB5, which inhibits PDGF-BB and PDGFR-β signaling^17^. A single intracavernous injection of APB5 (5 μg and 10 μg in 20 μl of PBS, respectively) significantly reduced the expression of PDGFR-β-positive pericytes in the penis of normal mice (Fig. 5a,b). Tube formation assay showed decreased branch points of MCPs exposed to APB5 (50 ng/ml) for 24 hours (Fig. 5c). The erectile function as determined by the ratios of maximal intracavernous pressure (ICP) and total ICP to mean systolic blood pressure were significantly lower in the normal mice 1 week after treatment with APB5 at doses of 5 μg and 10 μg than in the untreated normal mice (Fig. 5d–f).

### HGF restores cavernous pericyte content and erectile function in diabetic mice

**Figure 6:**
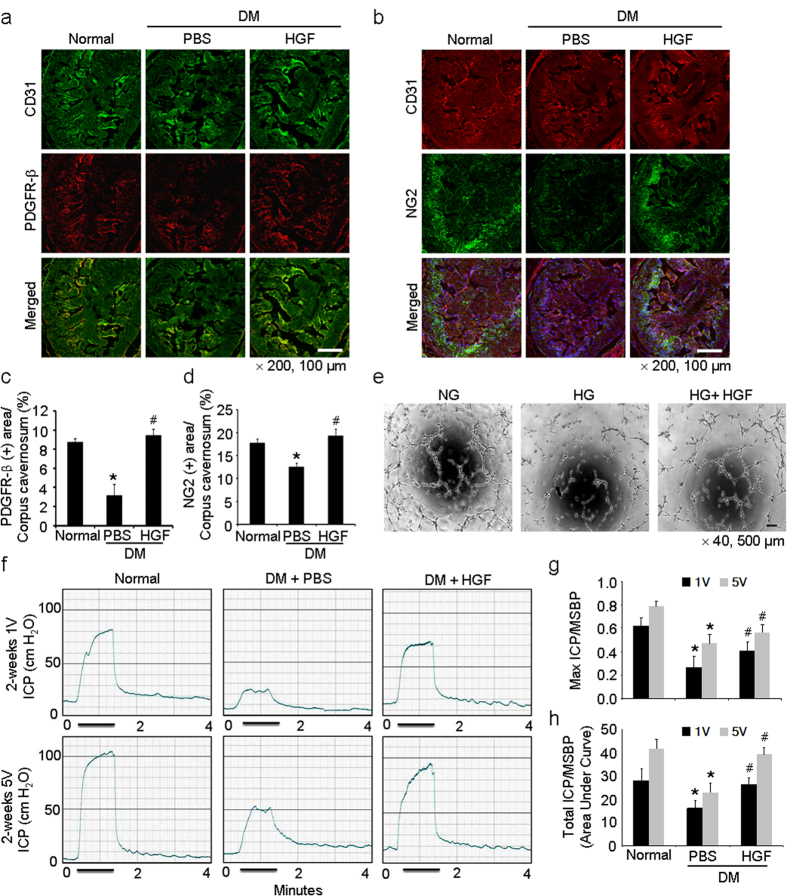
HGF protein transfer increases cavernous pericyte content and restores erectile function in diabetic mice. (**a**, **b**) Immunofluorescent double staining of cavernous tissue with antibodies to CD31 (green) and PDGFR-β (red) or CD31 (green) and NG2 (red) in age-matched controls or diabetic mice 2 weeks after receiving repeated intracavernous injections of PBS (days -3 and 0; 20 μl) or HGF protein (days -3 and 0; 4.2 μg/20 μl). Scale bar = 100 μm and screen magnification = ×200. (**c**, **d**). An image analyzer was used to quantitate the PDGFR-β and NG2-immunopositive area, respectively. Each bar depicts the mean ± standard deviation from *n* = 6 animals per group. *P < 0.01 vs. normal group and #*P *< 0.01 vs. PBS-treated diabetic group. (**e**) Tube formation assay in MCPs exposed to normal glucose condition (NG, 5 mmol), high-glucose condition (HG, 30 mmol), and high-glucose condition co-treated with rh-HGF (100 ng/ml) for 48 hours. Scale bars = 500 μm and screen magnification = ×40. (**f**) Representative intracavernous pressure (ICP) responses in each group. The cavernous nerve was stimulated at 1 and 5 V. The stimulus interval is indicated by a solid bar. **(g**, **h)** Ratios of mean maximal ICP and total ICP (area under the curve) to mean systolic blood pressure (MSBP) were calculated for each group. Each bar depicts the mean ± standard deviation from *n* = 6 animals per group. *P < 0.05 vs. normal group and #*P* < 0.05 vs. PBS-treated diabetic group. DM = diabetes mellitus; HGF = hepatocyte growth factor; PDGFR-β, platelet-derived growth factor receptor-beta.

We examined the effect of rh-HGF on cavernous pericyte expression in diabetic mice. HGF is known as a potent angiogenic factor and promotes recruitment of pericytes and SMCs toward ECs^18^,^19^. Repeated intracavernous injections of rh-HGF protein (days -3 and 0; 4.2 μg/20 μl of PBS) completely restored NG2- and PDGFR-β-positive pericyte contents in the diabetic mice 2 weeks after treatment (Fig. 6a–d). Tube formation assay showed decreased branch points of MCPs exposed to high glucose condition, which were completely recovered by treatment with rh-HGF protein (100 ng/ml) for 48 hours (Fig. 6e). Next, we observed that repeated intracavernous injections of the rh-HGF protein restored erection parameters (maximal and total ICP) in diabetic mice, which reached up to 85% to 100% of control values (Fig. 6f–h).

### HGF decreases cavernous permeability in diabetic mice and in MCPs-MCECs co-culture system

**Figure 7:**
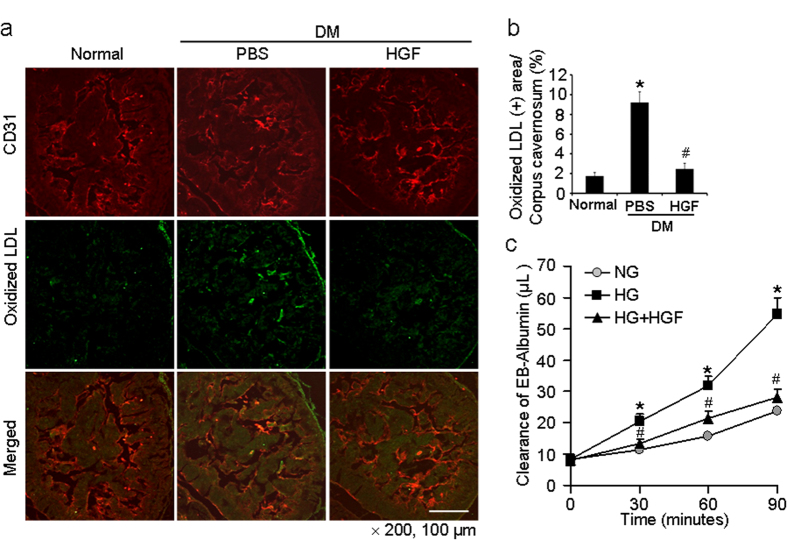
HGF protein transfer decreases cavernous permeability in diabetic mice in vivo and in pericytes-endothelial cell co-culture system in vitro. (**a**) Immunofluorescent double staining of cavernous tissue with antibodies to CD31 (green) and oxidized LDL (red) in age-matched controls or diabetic mice 2 weeks after receiving repeated intracavernous injections of PBS (days -3 and 0; 20 μl) or HGF protein (days -3 and 0; 4.2 μg/20 μl). Scale bar = 100 μm and screen magnification = ×200. (**b**) An image analyzer was used to quantitate oxidized-LDL immunopositive area. Each bar depicts the mean ± standard deviation from *n* = 6 animals per group. *P < 0.01 vs. normal group and #*P* < 0.01 vs. PBS-treated diabetic group. (**c**) Effect of HGF on high glucose-induced pericyte-endothelial cell permeability. Pericytes and endothelial cells were cultured and treated under the following conditions: the cells exposed to normal glucose condition (NG, 5 mmol), high-glucose condition (HG, 30 mmol), and high-glucose condition (30 mmol) co-treated with rh-HGF (100 ng/ml) for 48 hours before addition of Evans blue-albumin (EBA) solution. Clearance of EBA was measured up to 90 minutes. Each bar depicts the mean ± standard deviation from six-independent experiments. *P < 0.001 vs. NG group. #*P* < 0.001 vs. HG group. DM = diabetes mellitus; HGF = hepatocyte growth factor; Oxidized LDL, oxidized low-density lipoprotein.

To determine the relationship between pericyte loss and vascular permeability, we determined extravasation of oxidized low-density lipoprotein (LDL) by immunohistochemical staining of penile tissue with antibodies to CD31 and oxidized LDL. We observed a significant leakage of oxidized LDL across the endothelial layer in penis of diabetic mice compared with control mice. The leakage was mainly noted in the subendothelial area throughout the corpus cavernosum. Repeated intracavernous injections of rh-HGF protein significantly decreased the extravasation of oxidized LDL in the penis of diabetic mice, which was comparable to the level found in age-matched controls (Fig. 7a,b).

We further determined the role of pericytes on cavernous permeability by using primary cultured mouse cavernous pericytes (MCPs)-endothelial cells (MCECs) co-culture system. The leakage of Evans blue was significantly higher in MCPs-MCECs exposed to the high-glucose condition than in the cells exposed to the normal glucose condition, which was returned to baseline level after treatment with rh-HGF protein (Fig. 7c).

## Discussion

Pericytes are known to be ubiquitously present in microvessels, such as capillaries, postcapillary venules, and terminal arterioles, although their presence is also reported in large blood vessels^20^. Microvessels consist of ECs that are covered by pericytes, whereas large blood vessels usually appear as layers of ECs, SMCs, and elastic and collagen fibers^21^,^22^. Similar to this finding, we observed that the distribution of pericytes was more prominent in the adjacent microvessels in the subtunical area and in the dorsal nerve bundle of mice than in cavernous sinusoids or large vessels, such as dorsal vein. In the cavernous artery and dorsal artery of mice, the endothelium was tightly covered with SMCs and was rarely associated with pericytes. Furthermore, we confirmed the presence of pericytes in human corpus cavernosum tissue and successfully isolated pericytes from mouse penis.

The pericyte density, defined as the ratio between ECs and pericytes, varies between organs and is highest in the central nervous system, in which vascular endothelial permeability is tightly regulated^23^. In the present study, the pericyte coverage was relatively lower in the cavernous sinusoids than in the microvessels of the subtunical area. Different from other vasculatures, the cavernous sinusoids are dynamically collapsed or distended during flaccid or tumescence status. We recently observed in mice that endothelial cell-cell junction proteins are more sparsely distributed in the endothelium of cavernous sinusoids than in the endothelium of other vessels, including coronary and femoral blood vessels, which explains the more dynamic nature of the cavernous sinusoids during physiologic erection than the vasculature from other parts of body^24^,^25^. In agreement with this finding, relatively low pericyte coverage in cavernous sinusoids may be helpful for the dynamic expansion of the sinusoids during penile erection.

The reason why pericytes are most abundantly distributed in the microvessels of the subtunical area is as yet unclear. Further studies are required to determine whether the pericyte-rich area may constitute a penile stem cell niche and whether cultured penile pericytes have lineage-specific differentiation or regeneration potential in damaged cavernous tissue from various causes. It is also necessary to document whether the subtunical pericyte-rich area is involved in active contraction of subtunical vessels to reduce blood flow out of the penis to sustain penile erection.

Pericyte loss or dropout has been noted in a variety of pathologic conditions, such as diabetic retinopathy, tumor, trauma, and sepsis^11^,^26^,^27^. Oxidized LDL-mediated oxidative stress is known to induce pericyte apoptosis and diabetic retinopathy^11^. Similar to this finding, we observed a significant leakage of oxidized LDL in the corpus cavernosum of diabetic mice with a concurrent decrease in pericyte content, which may induce cavernous inflammation and fibrosis and impede the expandability of erectile tissue.

Next, we investigated whether loss of pericyte function in the penis by use of the APB5 would deteriorate erectile function in normal mice. Previous study showed that the inhibition of the PDGF-BB and PDGFR-β pathway results in pericyte dysfunction^28^. And pericytes, interacting with endothelial cells, were found to have an active role in vascular tube formation or vessel formation^29^. In the present study, tube formation assay showed that APB5 significantly inhibits vascular or sinusoidal tube formation by cavernous pericytes, which in turn results in erectile dysfunction. Thus, cavernous pericyte might be an important cellular regulator in physiologic erection.

Finally, we checked whether the restoration or reinforcement of pericyte function could induce recovery of erectile function in pathologic conditions, such as diabetes mellitus. HGF is a potent angiogenic factor and is also well-known to induce recruitment of pericytes^18^,^19^. In the present study, repeated intracavernous injections of rh-HGF protein completely restored pericyte content and significantly decreased oxidized LDL leakage, which induced recovery of erectile function. Moreover, rh-HGF also decreased the leakage of Evans blue in high-glucose condition in MCPs-MCECs co-culture system. Therefore, these indicate that cavernous pericyte has a definite role to regulate the permeability between blood and sinusoidal or vascular endothelial layer.

To the best of our knowledge, this is the first study showing the differential distribution of pericytes in the corpus cavernosum in the mouse and human and documenting the functional significance of pericytes in physiologic and pathologic conditions. Pericyte gain- and loss-of-function studies indicate that the structural and functional integrity of pericytes are critical for maintaining penile erection by regulation of the permeability of cavernous endothelial layer, although other roles of pericytes remain to be investigated. Through the present study, we believe that our results will open new avenues of exploration in the field of erectile function/dysfunction in association with pericyte pathobiology and the development of new therapeutics targeting pericyte function.

## Methods

Methods details are given in the Supplemental information online.

### Animals and preparation of human corpus cavernosum tissue

Male C57BL/6J mice were used and randomly grouped in this study. The experiments were approved by the institutional animal care and use subcommittee of our university. Human corpus cavernosum tissues were obtained from patients with congenital penile curvature who have normal erectile function (*n* = 4; mean 25.5 years) during reconstructive penile surgery. All tissue donors provided informed consent, and the experiments were approved by the internal review board of our university. Animal care and all experimental procedures were conducted in accordance with the approval and guidelines of the INHA Institutional Animal Care and Use Committee (INHA IACUC) of the Medical School of Inha University.

### Nerve-mediated erection studies

Nerve-mediated erection studies were performed as described in the Supplemental information online.

### Immunohistochemistry and 3D reconstruction

Detail immunohistochemistry and 3-D reconstruction was performed as described in the Supplemental information online. (see Supplemental Fig. 1 online).

### Isolation and culture of mouse cavernous pericytes

Primary culture of MCPs and *in vitro* tube formation assay, permeability assay were performed as described in Supplemental information online.

### Statistical analysis

Results are expressed as means ± standard deviations. Statistical analysis was performed by using Mann-Whitney *U*-tests or one-way analysis of variance. Probability values less than 5% were considered significant. We performed statistical analyses by using SigmaStat 3.11 software (Systat Software Inc., Richmond, CA, USA).

## Additional Information

**How to cite this article**: Yin, G. N. *et al.* The pericyte as a cellular regulator of penile erection and a novel therapeutic target for erectile dysfunction. *Sci. Rep.* **5**, 10891; doi: 10.1038/srep10891 (2015).

## Supplementary Material

Supplementary Information

Supplementary Video File 1

Supplementary Video File 2

Supplementary Video File 3

Supplementary Video File 4

Supplementary Video File 5
