# Supplementary material for: The pericyte as a cellular regulator of penile erection and a novel therapeutic target for erectile dysfunction
Source: Sci Rep. 2015 Jun 5;5:10891. doi: 10.1038/srep10891 (PMC4456662; doi:10.1038/srep10891)
Supplement: Supplementary Information [file srep10891-s1.pdf]

Supplementary Information

The pericyte as a cellular regulator of penile erection and a novel therapeutic target for erectile dysfunction

Guo Nan Yin[1,*], Nando Dulal Das[1,*], Min Ji Choi[1], Kang-Moon Song[1], Mi-Hye Kwon[1], Jiyeon Ock[1], Anita Limanjaya[1], Kalyan Ghatak[1], Woo Jean Kim[1], Jae Seog Hyun[2], Gou Young Koh[3], Ji-Kan Ryu[1,4,†], and Jun-Kyu Suh[1,†]

[1]National Research Center for Sexual Medicine and Department of Urology, Inha University School of Medicine, Incheon 400-711, Republic of Korea

[2]Department of Urology, Gyeongsang National University School of Medicine, Jinju 660-702, Republic of Korea

[3]Department of Biological Sciences and Laboratory for Vascular Biology, Korea Advanced Institute of Science and Technology (KAIST), Daejeon 305-701, Republic of Korea

[4]Inha Research Institute for Medical Sciences, Inha University School of Medicine, Incheon 400-711, Republic of Korea

[*] Guo Nan Yin and Nando Dulal Das contributed equally to this study.

[†]Corresponding author: Jun-Kyu Suh, MD, PhD or Ji-Kan Ryu, MD, PhD

Jun-Kyu Suh, MD, PhD

National Research Center for Sexual Medicine and Department of Urology

Inha University School of Medicine

7-206, 3rd ST, Shinheung-Dong, Jung-Gu, Incheon 400-711

Republic of Korea

Tel: 82-32-890-3441

Fax: 82-32-890-3097

E-mail: jksuh@inha.ac.kr

Ji-Kan Ryu, MD, PhD

National Research Center for Sexual Medicine, Department of Urology, and Inha Research Institute for Medical Sciences

Inha University School of Medicine

7-206, 3rd ST, Shinheung-Dong, Jung-Gu, Incheon 400-711

Republic of Korea

Tel: 82-32-890-3505

Fax: 82-32-890-3099

E-mail: rjk0929@inha.ac.kr

Supplemental Methods

Animals and treatment

Male C57BL/6J mice were used and randomly grouped in this study. The experiments were approved by the institutional animal care and use subcommittee of our university. Diabetes was induced in 8-week-old mice by intraperitoneal injections of streptozotocin (50 mg/kg) for 5 days consecutively as we previously described[1]. At 8 weeks after the induction of diabetes, animals were anesthetized with ketamine (100 mg/kg) and xylazine (5 mg/kg) intramuscularly, and penis was exposed by use of sterile technique. The diabetic mice received repeated intracavernous injections of phosphate-buffered saline (PBS, days -3 and 0; 20 μl) or recombinant human-hepatocyte growth factor (rh-HGF) protein (days -3 and 0; 4.2 μg/20 μl of PBS), and normal mice received a single intracavernous injection of APB5 (1 μg, 5 μg, or 10 μg/20 μl of PBS), an anti-platelet-derived growth factor receptor-beta (PDGFR-β) blocking antibody. At 2 weeks after intracavernous injection of rh-HGF and 1 week after intracavernous injection of APB5, erectile function was measured during electrical stimulation of the cavernous nerve. The penis was then harvested for histologic examination. Fasting and postprandial blood glucose levels were determined with an Accu-Check blood glucose meter (Roche Diagnostics, Mannheim, Germany) before the mice were sacrificed (data not shown).

Immunohistochemistry and 3D reconstruction

The mouse or human penis tissue was fixed in 4% paraformaldehyde for 24 hours at 4°C, and frozen tissue sections (7-μm [thin-cut] or 50-μm [thick-cut]) were incubated with antibodies to CD31 (an endothelial cell marker; BD Biosciences, Bedford, MA, USA; 1:50), Von willebrand factor (VWF, an endothelial cell marker: Santa Cruz Biotechnology, Santa Cruz, CA, USA; 1:50) fluorescein isothiocyanate (FITC)-conjugated antibody to smooth muscle α-actin (a smooth muscle cell marker; Sigma-Aldrich, St. Louis, MO, USA; 1:200), PDGFR-β (a pericyte marker;

Santa Cruz; 1:50), NG2 chondroitin sulfate proteoglycan (Millipore, San Francisco, CA, USA; 1:50), or oxidized low-density lipoprotein (oxidized LDL, Abcam, Cambridge, MA, USA; 1:400) at 4°C overnight. After several washes with PBS, the sections were incubated with tetramethyl rhodamine isothiocyanate (TRITC)- (Zymed Laboratories, South San Francisco, CA, USA) or FITC-conjugated secondary antibodies (Molecular Probes Inc., Eugene, OR, USA) for 2 hours at room temperature. The **Supplementary Figure 1** shows schematic principles of 3-D reconstruction. To visualize the internal penile structures, both transverse and longitudinal sections were floated and coverslipped and finally subjected to 3-D reconstruction from stacks of 2-D images. Signals were visualized and digital images were obtained with a confocal microscope (FV1000, Olympus, Tokyo, Japan). Quantitative analysis of histologic examinations was done with an image analyzer system (National Institutes of Health [NIH] Image J 1.34, http://rsbweb.nih.gov/ij/).

Nerve-mediated erection studies

We evaluated erectile function (n = 6 per group) by electrical stimulation of the cavernous nerve 2 weeks after rh-HGF treatment and 1 week after APB5 treatment. Bipolar platinum wire electrodes were placed around the cavernous nerve. Stimulation parameters were 1 to 5 V at a frequency of 12 Hz, a pulse width of 1 ms, and a duration of 1 minute. During tumescence, the maximal intracavernous pressure (ICP) was recorded. The total ICP was determined by the area under the curve from the beginning of cavernous nerve stimulation to a point 20 seconds after stimulus termination. Systemic blood pressure was measured with a noninvasive tail-cuff system (Visitech Systems, Apex, NC, USA). The ratios of maximal ICP and total ICP to mean systolic blood pressure (MSBP) were calculated to normalize for variations in systemic blood pressure.

Cell culture

The mouse cavernous pericytes (MCPs) were prepared and maintained as previously described with minor modifications[2]. Briefly, eight-week-old C57BL/6J mice were used in this study. A schematic diagram of the procedure used to isolate the MCPs is illustrated in **Figure 3A**. Penis tissue was harvested and transferred into sterile vials containing Hank's balanced salt solution (GIBCO, Carlsbad, CA, USA) and was washed two times in PBS. The glans penis, urethra, and dorsal neurovascular bundle were removed from the penis, and only the corpus cavernosum tissue was used for primary pericyte culture. The corpus cavernosum tissue was cut into several pieces (1 mm) and the fragmented pieces of cavernous samples were settled by gravity into collagen I-coated 35-mm cell culture dishes (BD Biosciences). After 30 minutes incubation at 37°C with 300 µl complement Dulbecco's modified Eagle Medium (DMEM, GIBCO), supplemented with 10% fetal bovine serum (FBS), 1% penicillin/streptomycin, and 10 nM human pigment epithelium-derived factor (PEDF; Sigma-Aldrich), we added 900 µl complement medium additionally and the samples were incubated at 37°C with 5% CO_2. The medium was changed each 2 days, after the cells were confluent and spread on the whole bottom of the dish (about 2 weeks after the start of culture). Only sprouting cells were used for subcultivation. The sprouting cells were seeded onto dishes coated with 50 µl/ml collagen I (Advanced BioMatrix, San Diego, CA, USA). Cells at passages between 2 and 3 were used for experiments.

To determine cell type, primary cultured cells were cultured on sterile cover glasses (Marienfeld Laboratory, Lauda-Königshofen, Germany) that were placed on the bottom of 24-well plates and grown until nearly confluent. The cells were washed three times with PBS and then fixed with 4% paraformaldehyde for 10 minutes at room temperature and incubated with blocking solution for 30 minutes at room temperature. Individual chambers were incubated with antibodies to NG2 chondroitin sulfate proteoglycan (Millipore; 1:50), PDGFR-β (Santa Cruz; 1:50), CD31 (BD Biosciences; 1:50), FITC-conjugated antibody to smooth muscle α-actin (Sigma-Aldrich; 1:200), or CD90 (a fibroblast marker; R&D Systems Inc., Minneapolis, MN,

USA; 1:50) overnight at 4°C in a moist chamber. After several washes with PBS, the chambers were incubated with FITC- or TRITC-conjugated secondary antibodies (Zymed) for 2 hours at room temperature. We used human brain microvascular pericytes (HBMP) as a positive control. Rat aorta smooth muscle cell line (A7r5) and mouse embryonic fibroblast cell line (NIH3T3) were used as negative controls. Signals were visualized and digital images were obtained with a confocal microscope (FV1000, Olympus, Tokyo, Japan).

The mouse cavernous endothelial cells (MCECs) were isolated and cultured as we previously described[3].

In vitro Tube formation assay

The tube formation assay was performed as previously described [3]. About 50 μl of growth factor-reduced Matrigel (Collaborative Biomedical Products) was dispensed into 96-well tissue culture plates at 4°C. After gelling at 37°C for at least 30 minutes, the conditioned MCPs were seeded onto the gel at 2×10^4 cells/well in 200 μl of M199 medium. The assay was performed in a CO_2 incubator and the plates were incubated at 37°C for 24 hours. Images were obtained with a phase-contrast microscope and the numbers of tubes in each well of the plate were counted at a screen magnification of ×40. Only branch points were counted.

In vitro permeability assay

To examine the role of MCPs and MCECs on cavernous vascular permeability, MCPs and MCECs were co-cultured in the Transwell filters (1.0 μm pore size, Becton Dickinson Labware, Franklin Lakes, NJ) and the permeability was assayed by measuring the leakiness of Evans blue (Sigma-Aldrich) bound to bovine serum albumin (BSA, Bovogen Biologicals, VIC, Australia) as previously described[4]. MCPs at a density of 1×10^5 cells/well were seeded on the bottom side of the insert and grown to confluence in Transwell filters. After 2 days, MCECs (1×10^5

cells/well) were added to the upper inserts and grown to confluence for other 3 days, as previously described[5].

To mimic an *in vivo* model for diabetes-induced pericyte-endothelial cell dysfunction, primary cultured MCPs-MCECs were serum-starved for 24 hours and were exposed to the normal-glucose (5 mmol, Sigma-Aldrich) or high-glucose (30 mmol) condition for 48 hours. In order to examine the effect of rh-HGF on cavernous vascular permeability in high-glucose condition, the MCPs and MCECs were cultured and treated under the following conditions: the cells exposed to normal glucose condition (5 mmol), the cells exposed to the high-glucose condition (30 mmol), and the cells exposed to the high-glucose condition (30 mmol) and treated with rh-HGF (100 ng/ml). The permeability was measured at selected time points as previously described[4].

References

1 Jin, H. R. et al. Intracavernous delivery of a designed angiopoietin-1 variant rescues erectile function by enhancing endothelial regeneration in the streptozotocin-induced diabetic mouse. *Diabetes* **60**, 969-980 (2011).

2 Neng, L. et al. Isolation and culture of endothelial cells, pericytes and perivascular resident macrophage-like melanocytes from the young mouse ear. *Nat. Protoc*. **8**, 709-720 (2013).

3 Yin, G. N. et al. Matrigel-based sprouting endothelial cell culture system from mouse corpus cavernosum is potentially useful for the study of endothelial and erectile dysfunction related to high-glucose exposure. *J. Sex. Med*. **9**, 1760-1772 (2012).

4 Richard, L. F., Dahms, T. E., &Webster, R. O. Adenosine prevents permeability increase in oxidant-injured endothelial monolayers. *Am. J. Physiol*. **274**, H35-42 (1998).

5 Wisniewska-Kruk, J. et al. A novel co-culture model of the blood-retinal barrier based on primary retinal endothelial cells, pericytes and astrocytes. *Exp. Eye. Res*. **96**, 181-190 (2012).

Supplementary Figure Legend

Supplemental Figure 1. The principle of three-dimensional reconstruction. Reconstruction of a three-dimensional image from stacks of two-dimensional images allows volume rendering and is useful for visualizing internal structures.

Supplemental Figure 2. Conversion of a set of two-dimensional images to a three-dimensional image in the penis of normal mice. Three-dimensional reconstructions of confocal image z-stacks of transverse sections (50 μm) depicted by CD31 (an endothelial cell marker, blue), smooth muscle α-actin (a smooth muscle cell marker, green), and NG2 staining (a pericyte marker, red) in a 12-week-old male mouse. **(a,b)** Cavernous sinusoids; **(c,d)** subtunical area; **(e,f)** dorsal vein; **(g,h)** dorsal artery and dorsal nerve bundle; **(i,j)** cavernous artery. Scale bar = 5, 10, 50, 50, and 20 μm, and screen magnification = ×3000, ×200, ×400, ×400, and ×1000 respectively for B, D, F, H, and J. Images are representative of four independent experiments. The live 3-D video images for each area are available in the *Online Supplementary Information*.

Supplementary Video File 1 - Live 3-D video for cavernous sinusoids stack; Screen magnification = ×3000

Supplementary Video File 2 - Live 3-D video for subtunical area stack; Screen magnification = ×1000

Supplementary Video File 3 - Live 3-D video for dorsal vein stack; Screen magnification = ×400

Supplementary Video File 4 - Live 3-D video for dorsal neurovascular bundles stack; Screen magnification = ×400

Supplementary Video File 5 - Live 3-D video for cavernous artery stack; Screen magnification = ×1000

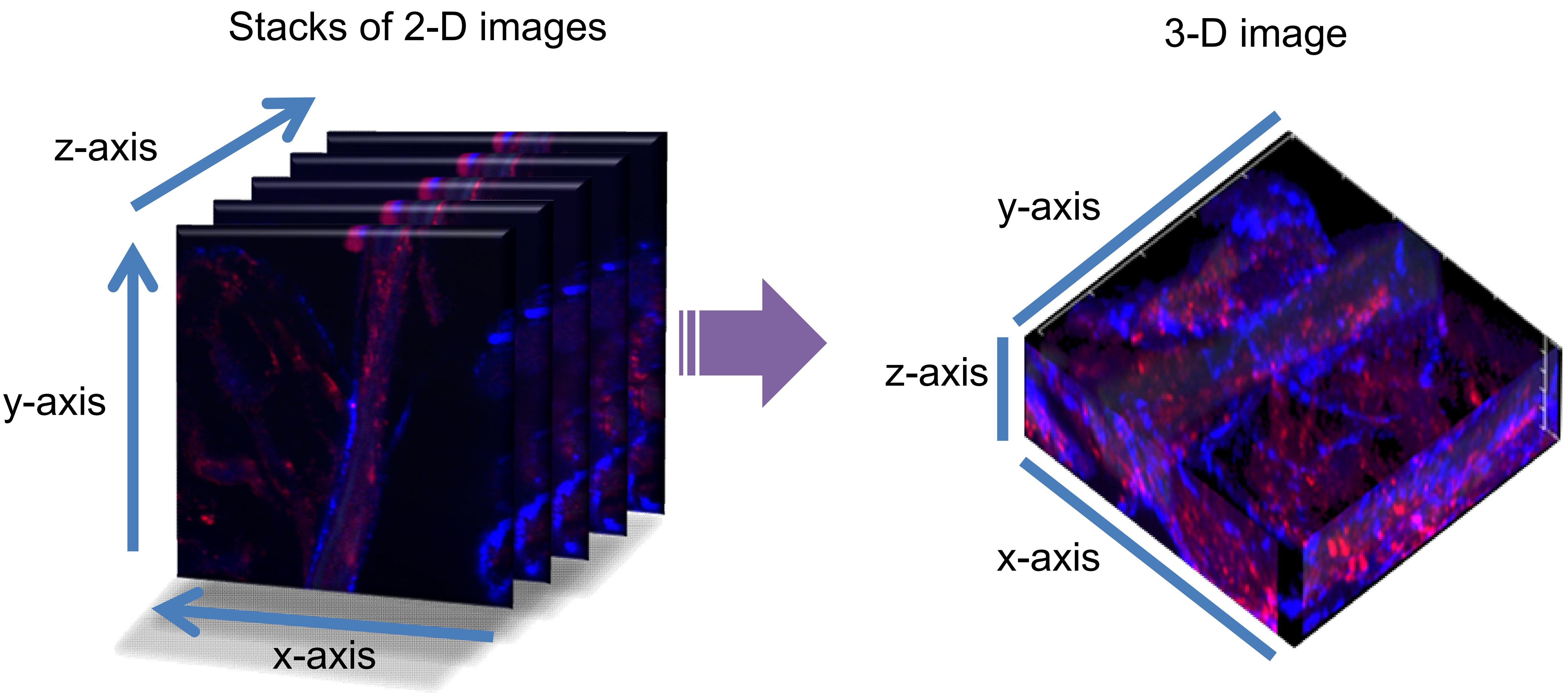

Supplemental Figure 1

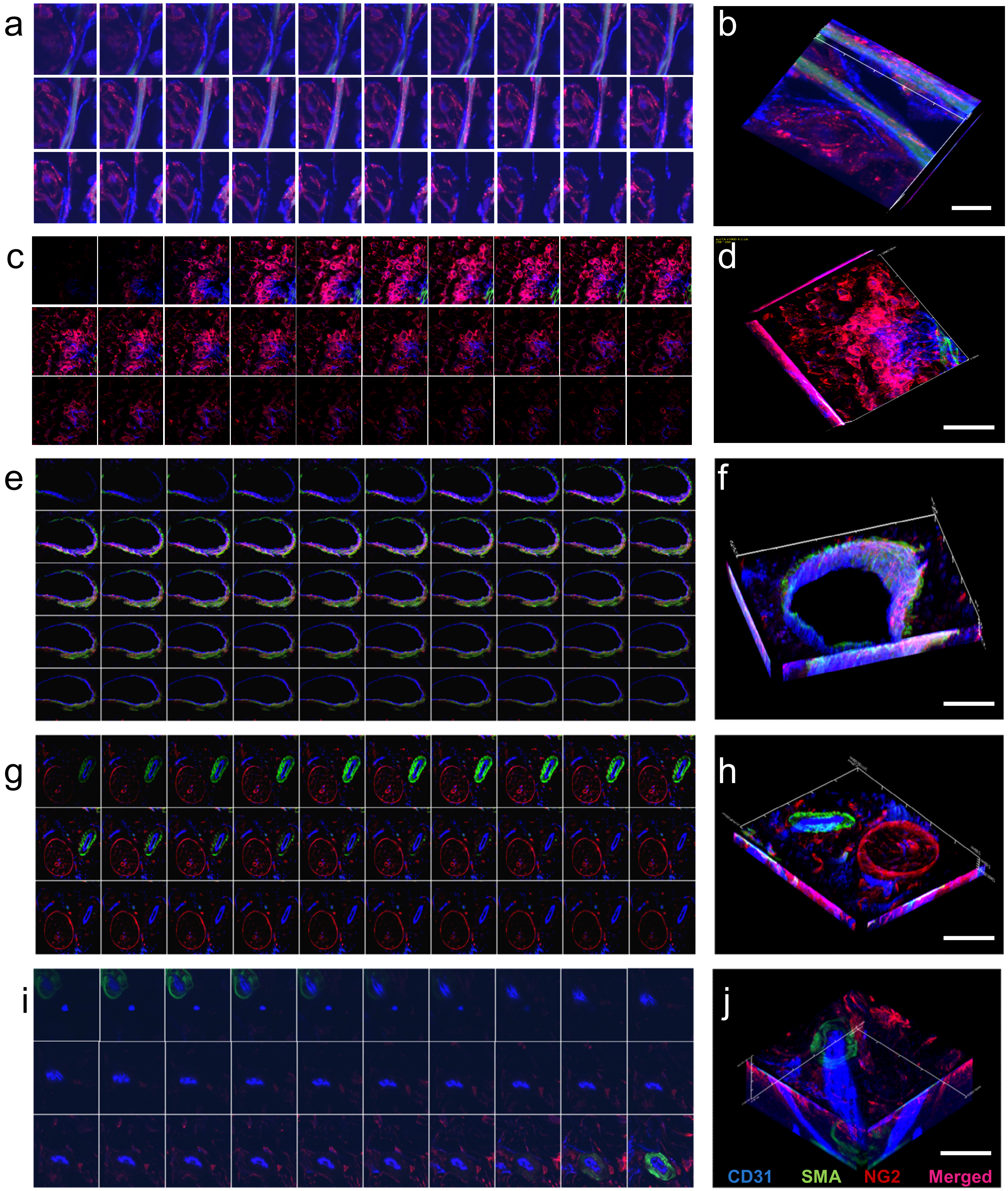

Supplemental Figure 2
